# Obesity paradox in patients undergoing lung lobectomy – myth or reality?

**DOI:** 10.1186/s12893-018-0395-2

**Published:** 2018-08-17

**Authors:** Lubomír Tulinský, Marcel Mitták, Hana Tomášková, Petr Ostruszka, Igor Penka, Peter Ihnát

**Affiliations:** 1 Department of Surgery, University Hospital Ostrava, 17.listopadu 1790, 708 52 Ostrava, Czech Republic; 2 Department of Surgical studies, Faculty of Medicine, University of Ostrava, Syllabova 19, 703 00 Ostrava, Czech Republic; 3 Department of Epidemiology and Public Health, Faculty of Medicine, University of Ostrava, Syllabova 19, 703 00 Ostrava, Czech Republic

**Keywords:** Obesity paradox, Body mass index, Pulmonary lobectomy, Postoperative morbidity

## Abstract

**Background:**

The aim of the present study was to evaluate the impact of BMI on the short-term outcomes of patients undergoing lung lobectomy.

**Methods:**

This was a retrospective clinical cohort study conducted in a single institution to assess the short-term outcomes of obese patients undergoing lung resection. Intraoperative and postoperative parameters were compared between the two study subgroups: obese (BMI ≥30 kg/m2) and non-obese patients (BMI < 30 kg/m2).

**Results:**

In total, 203 patients were enrolled in the study (70 obese and 133 non-obese patients). Both study subgroups were comparable with regards to demographics, clinical data and surgical approach (thoracoscopy vs. thoracotomy). The surgery time was significantly longer in obese patients (*p* = 0.048). There was no difference in the frequency of intraoperative complications between the study subgroups (*p* = 0.635).

The postoperative hospital stay was similar in both study subgroups (*p* = 0.366). A 30-day postoperative morbidity was higher in a subgroup of non-obese patients (33.8% vs. 21.7%), but the difference was not significant (*p* = 0.249). In the subgroup of non-obese patients, a higher frequency of mild and severe postoperative complications was observed. However, the differences between the study subgroups were not statistically significant due to the borderline *p*-value (*p* = 0.053). The 30-day postoperative mortality was comparable between obese and non-obese patients (*p* = 0.167).

**Conclusions:**

Obesity does not increase the incidence and severity of intraoperative and postoperative complications after lung lobectomy. Slightly better outcomes in obese patients indicate that obesity paradox might be a reality in patients undergoing lung resection.

## Background

Lung cancer is the most common cause of cancer-related death worldwide and the most common cancer among men in terms of incidence (1.82 million new cases globally, and 1.56 million of deaths) [1]. Surgical resection presents the gold standard of treatment for patients with non–small-cell lung cancer. After lung resection, several risk factors for the development of postoperative complications have been identified – age, smoking, obstructive pulmonary disease, cardiovascular disease, and ASA score [2, 3].

Obesity affects 10–30% of adults in European countries and presents the greatest pandemic of the twenty-first century. Excessive weight is a significant risk factor for intraabdominal perioperative complications, surgical site infections (SSI) and incisional hernias after abdominal surgery [4–6]. Obesity also significantly increases the risk of postoperative complications like myocardial infarction, peripheral nerve injury, and respiratory or urinary tract infection [5]. However, the association between Body Mass Index (BMI) and postoperative complications after lung resection has not been investigated sufficiently till now.

Within the last few years, authors of several studies suggested that the risk of postoperative complications after cardiothoracic surgery in obese patients may be similar to non-obese patients [7–9]. Some authors have even observed better postoperative outcomes in overweight and obese patients after cardiothoracic surgery – so called *obesity paradox* [10–13]*.*

To the best of our knowledge, relevant data regarding the relationship between BMI and short-term outcomes of lung resection are insufficient in the available literature. The aim of the present study was to investigate the impact of BMI on early postoperative outcomes in patients undergoing lung lobectomy.

## Methods

### Study design

This was a retrospective clinical cohort study performed at University Hospital Ostrava (Czech Republic) over a 3-year study period (1st January 2014 – 31st December 2016). All patients who underwent pulmonary lobectomy due to lung cancer or benign pulmonary lesion were considered for eligibility of the study. Exclusion criteria were inflammatory pulmonary tumors, emergency procedures and patients with incomplete data.

All patients were divided into two subgroups: obese (BMI ≥30) and non-obese patients (BMI < 30). The BMI was used as an objective measure of obesity. The cut-off BMI of 30 was defined in accordance with the official WHO classification.

The basic demographic and clinical data were extracted from medical records. The following parameters were analyzed: age, sex, BMI, American Society of Anesthesiologists (ASA) classification, tumor size and histopathology, perioperative outcomes (surgery time, blood loss, conversions), and short-term postoperative outcomes (length of hospital stay, 30-day postoperative morbidity and mortality).

Postoperative complications were graded according to the Clavien-Dindo classification system modified for thoracic surgery, which was introduced by Seely et al. in 2010 [14]. Prolonged postoperative air leak was defined as an air leak lasting ≥7 days. Grade II complications (prolonged air leak, pneumothorax and emphysema) did not require any surgical intervention or specific treatment. Grade III complications were managed via reinsertion of chest tube or via surgical revision with/without general anesthesia.

### Surgical technique

In all study patients, pulmonary lobectomy was performed after standard antibiotic and antithrombotic prophylaxis. The type of surgical access (thoracotomy, thoracoscopy) was determined by preoperative staging. The conversion of minimally invasive approach to thoracotomy was performed due to intraoperative complications or technical problems. All patients underwent anatomic pulmonary lobectomy and mediastinal lymph node dissection under general anesthesia with single-lung ventilation using dual-lumen endotracheal tubes. The technical details of conventional and thoracoscopic lobectomy have been described in detail elsewhere [15, 16].

### Statistical analysis

The acquired data underwent analysis by means of descriptive statistics. For the evaluation, a non-parametric Wilcoxon test was used. The chi-square test or Fisher’s exact test was used to evaluate the differences in the categories. A level of significance of a 0.05 and *P* values < 0.05 was considered statistically significant.

## Results

In total, 203 patients after pulmonary lobectomy were included in the study. The basic demographics and clinical data of study patients are presented in Table 1. There were 77 (37.9%) women and 126 (62.1%) men; the mean age was 64.4 ± 9.1 years (ranging from 25 to 84 years). There were 111 (54.6%) patients preoperatively classified as ASA II, and 84 (41.4%) patients classified as ASA III. The mean tumor size was 3.7 ± 2.3 cm; adenocarcinoma and spinocellular carcinoma were the most frequent lung tumors.

**Table 1:** Demographics and clinical data of study patients

	BMI < 30 (*n* = 133)	BMI ≥30 (*n* = 70)	*p*-value	Total (*n* = 203)
Age (years, mean ± SD)	64.0 ± 9.2	65.2 ± 8.9	0.211	64.4 ± 9.1
Gender, n (%)				
Female	51 (38.4)	26 (37.1)	0.867	77 (37.9)
Male	82 (61.6)	44 (62.9)		126 (62.1)
BMI (kg/m2), mean ± SD)	25.0 ± 3.4	34.1 ± 4.0	< 0.001	28.2 ± 5.7
ASA, n (%)				
I	2 (1.5)	2 (2.9)	0.073	4 (2)
II	79 (59.4)	32 (45.7)		111 (54.6)
III	48 (63.1)	36 (51.4)		84 (41.4)
IV	4 (3)	0 (0)		4 (2)
Tumour size (cm, mean ± SD)	3.8 ± 2.4	3.5 ± 1.9	0.092	3.7 ± 2.3
Histopathology findings, n (%)				
Adenocarcinoma	60 (45.1)	25 (35.7)	0.463	85 (41.9)
Spinocellular carcinoma	37 (27.8)	27 (38.6)		64 (31.5)
Neuroendocrine carcinoma	5 (3.8)	7 (10.0)		12 (5.9)
Parvocellular carcinoma	5 (3.8)	1 (1.4)		6 (3.0)
Sarcoma, Lymphoma	3 (2.3)	2 (2.8)		5 (2.5)
Metastasis	7 (5.3)	1 (1.4)		8 (3.9)
Benign lesion	16 (12.0)	7 (10.0)		23 (11.3)

The mean BMI in our study group was 28.2 ± 5.7 kg/m^2^ (ranging from 15 to 53 kg/m^2^). There were 70 (34.5%) patients with BMI ≥30 kg/m^2^ (classified as obese patients), and 133 (65.5%) non-obese patients (BMI < 30 kg/m^2^). The vast majority of obese patients had light to moderate degree of obesity (mean BMI of patients within obese study subgroup was 34.1 ± 4.0 kg/m2). There were six (2.9%) patients with morbid obesity (BMI > 40 kg/m^2^) in our study group. There were no statistically significant differences between study subgroups (obese vs. non-obese patients) with regards to age, gender, ASA classification, tumor size, and histopathology findings (Table 1).

The short-term outcomes of our study patients are presented in Table 2. Out of 203 lung lobectomies, minimally invasive (thoracoscopic) approach was employed in 77 (37.9%) patients; lung resection via anterolateral thoracotomy was performed in 126 (62.1%) patients. Both study subgroups were comparable with regard to surgical approach (*p* = 0.437).

**Table 2:** Intraoperative and postoperative outcomes of study patients

	BMI < 30 (*n* = 133)	BMI ≥30 (n = 70)	*p*-value	Total (*n* = 203)
Surgical approach, n (%)				
Thoracoscopy	53 (39.8)	24 (34.3)	0.437	77 (37.9)
Thoracotomy	80 (60.2)	46 (65.7)		126 (62.1)
Surgery time (min, mean ± SD)	95.2 ± 30.9	105.5 ± 32.2	0.048	98.7 ± 31.7
Operative blood loss (n, %)				
< 300 ml	129 (97.0)	67 (95.7)	0.635	196 (96.6)
≥ 300 ml	4 (3.0)	3 (4.3)		7 (3.4)
Hospital stay (days, mean ± SD)	11.3 ± 6.3	10.5 ± 5.7	0.366	11.0 ± 6.1
30-day postoperative morbidity,n (%)	45 (33.8)	19 (27.1)	0.249	64 (31.5)
Postoperative complications, n (%)				
1	9 (6.8)	9 (12.9)	0.053	18 (8.9)
2	24 (18.0)	6 (8.6)		30 (14.8)
3	12 (9.0)	4 (5.7)		16 (7.9)
4	0 (0.0)	0 (0.0)		0 (0.0)
5 (postoperative mortality)	5 (3.8)	0 (0.0)	0.167	5 (2.5)

The mean surgery time was 98.7 ± 31.7 min (range 30–190 min). The surgery time in a subgroup of obese patients was significantly longer than the surgery time of non-obese patients (*p* = 0.048).

Bleeding presented the only type of intraoperative complication observed in our study group. Clinically significant intraoperative blood loss (≥300 ml) was noted in 3 (4.3%) patients; the difference in the frequency of intraoperative complications between study subgroups was not significant (*p* = 0.635).

The mean duration of postoperative hospital stay was 11.0 ± 6.1 days (ranging from 4 to 35 days); the difference between study subgroups was not significant (*p* = 0.366).

**Fig. 1:**
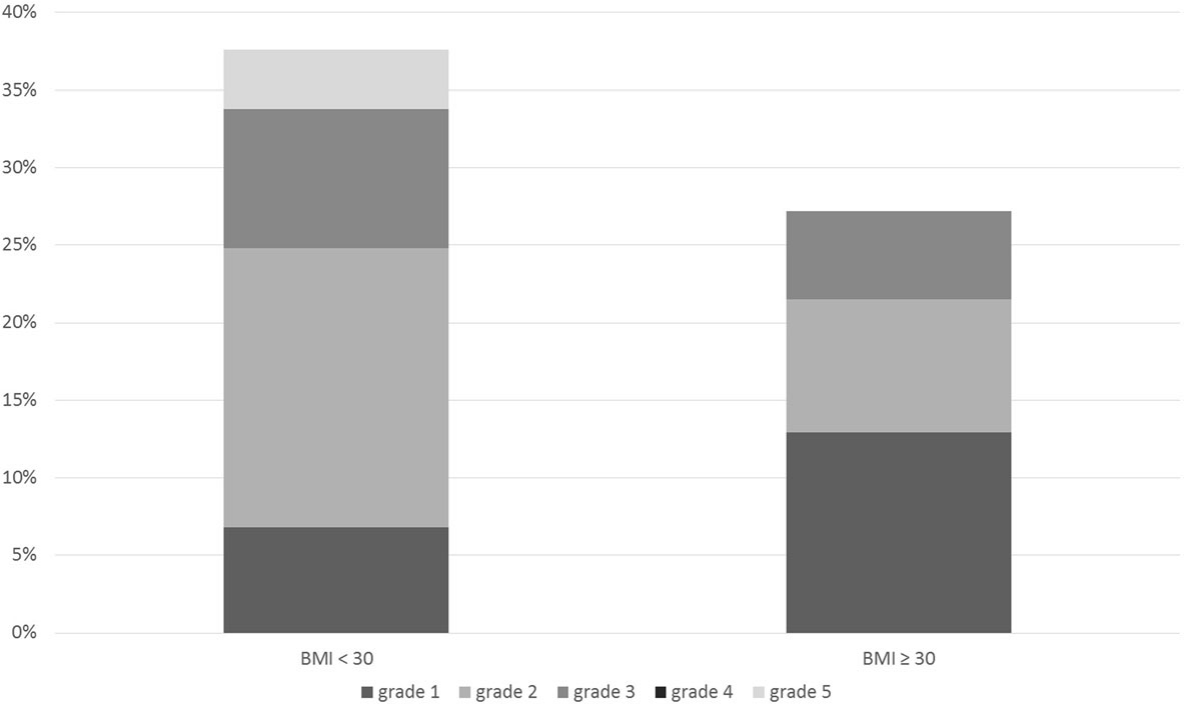
Severity of postoperative complications in study subgroups (Clavien-Dindo classification modified for thoracic surgery)

A 30-day postoperative morbidity detected in our study was 31.5%. A higher incidence of postoperative complications was noted in the subgroup of non-obese patients (33.8% vs. 27.1%), but the difference was not statistically significant (*p* = 0.249). According to Clavien-Dindo classification modified for thoracic surgery, mild postoperative complications (grade 1–2) were noted in 48 (23.6%) patients and severe complications (grade 3–5) in 21 (10.3%) patients. The distribution of postoperative complications according to their severity in both study subgroups is presented in Fig. 1. In the subgroup of non-obese patients, a higher incidence of mild and severe postoperative complications was observed. However, the differences between study subgroups were not statistically significant (due to the calculated borderline *p*-value).

The 30-day postoperative mortality in our study was 2.5%; the difference between study subgroups was not statistically significant (*p* = 0.167).

## Discussion

Obese patients represent an expanding and high-risk group of surgical patients, particularly with respect to wound and septic complications. Although BMI is a significant risk factor for perioperative and postoperative complications after intraabdominal surgery, data regarding the relationship between obesity and short-term outcomes of cardiothoracic surgery remain controversial [7–9, 12, 13].

The term “reverse epidemiology” has been proposed to address the apparently different relationship (contrary to general findings in the otherwise healthy population) between some risk factors and treatment outcomes – such as better dialysis outcomes in patients with obesity, hypertension or high cholesterol [17]. “Reverse epidemiology” findings regarding obese patients are termed obesity paradox (suggesting that obesity may be protective in some patients).

The obesity paradox was first described in 1999 among obese patients undergoing hemodialysis [18]; subsequently it was analyzed by many authors mainly in cardiology [10, 19]. In 2002, Gruberg et al. published better outcomes in moderately obese patients with coronary heart disease undergoing percutaneous coronary intervention [10]. Several meta-analyses proved obesity paradox as a viable theory in obese patients with heart failure and myocardial infarction [19, 20].

Vemmos et al. prospectively investigated 2785 patients after stroke over a period of 16 years [20]. Overweight and obese stroke patients had significantly lower mortality compared to non-obese patients. Stein et al. reported that obese patients with pulmonary embolism had lower hospital mortality than normal-weight subjects [21]. The same conclusions were drawn by Barba et al. in patients with acute venous thromboembolism [22].

There are several studies suggesting obesity paradox among patients after cardiac surgery [12, 13, 23]. However, data regarding the relationship between BMI and short-term outcomes of lung resections are very insufficient in the available literature.

Intraoperative results of our study group suggest that obese patients do not have an increased risk of perioperative complications during lung lobectomy – clinically significant intraoperative blood loss was comparable in both study subgroups. The surgery time of obese patients was significantly longer, but increased BMI did not affect the employed surgical approach (lobectomy performed by thoracoscopy vs. thoracotomy). The association between obesity and prolonged surgery time of lung lobectomy has also been reported by other authors. Julien et al. analyzed data from the database of the society of thoracic surgeons (19,337 patients included). The increased BMI was associated with longer surgery time, but was not associated with significantly higher 30-day mortality. On the contrary, BMI had an inverse association with duration of hospital stay [24].

In our study, there was no difference in outcomes regarding surgical approach of obese and non-obese patients. Owing to that fact, we conclude that obesity does not impact the technical feasibility and safety of thoracoscopic lung lobectomy. Currently, videothoracosopic access presents a standard surgical approach for lung lobectomy. Several recent studies demonstrate the same short-term outcomes of lung lobectomy performed through minimally invasive (thoracoscopy) and thoracotomy approach [15, 25]. According to our results, thoracoscopic lung resection can be performed safely in obese patients.

Postoperative outcomes of our study patients suggest that obesity is not a risk factor increasing postoperative morbidity/mortality after lung lobectomy. It seems that obese patients may even experience lower numbers of mild and severe postoperative complications. Although our data did not prove obesity paradox statistically, the borderline *p*-value implies that obesity paradox may be a reality in patients undergoing lung lobectomy.

Obese patients are more likely to have significant impairments of pulmonary gas exchange and respiratory mechanics during general anesthesia [26, 27]. In general, functional residual capacity (FRC) is decreased and intraabdominal pressure is higher in obese patients. Pelosi et al. demonstrated that with increasing BMI, lung compliance and FRC are decreasing, the resistance of the total respiratory system is increasing and the work of breathing is increasing [28]. Despite respiratory function impairments, obese patients after lung lobectomy show comparable or even better postoperative outcomes in comparison with non-obese patients [29, 30]. This underlines the fitting of the term “obesity paradox” for these patients.

Data regarding the impact of obesity on postoperative outcomes of patient after lung resection are hardly insufficient ion the available literature. Smith et al. reviewed the general thoracic surgery database and identified 499 patients after anatomic pulmonary resections (segmentectomies, lobectomies, bilobectomies, pneumonectomies). In this paper, no differences in the incidence of perioperative complications, postoperative morbidity and mortality between obese and non-obese patients were proved. Interestingly, authors observed a significant trend toward a protective effect of obesity on postoperative respiratory complications compared between non-obese and non-underweight patients [29].

Petrella et al. investigated a study group of 154 patients after standard pneumonectomy [31]. In contrast to our outcomes as well as the conclusions of the Smith’s study, fivefold more frequent postoperative respiratory complications were identified between overweight and obese patients. No differences between obese and non-obese patients were observed regarding cardiac complications and a 30-day mortality rate. Authors concluded that obesity should be considered an additional risk factor in patients requiring pneumonectomy.

Launer et al. published a paper with retrospective analysis of hospital discharge data from the largest database of inpatient care in the United States [30]. In total, the outcomes of 1238 obese and 31,983 non-obese patients after lung lobectomy due to cancer were analyzed. With regards to postoperative morbidity and mortality, there were no statistically significant differences between obese and non-obese patients. Authors therefore conclude that obesity should not be considered a risk factor of pulmonary resection.

The rationale of obesity paradox has not been clarified sufficiently till now. Childers and Allison used a mathematical model (U-shaped curve), explaining the main phenomena of reverse epidemiology [32]. Patients with extreme BMI values (severe underweight, morbid obesity) have highest mortality rates; patients with intermediate BMI values (overweight, light or moderate obesity) have the lowest mortality rates. In our study group, the vast majority of obese patients had a light to moderate degree of obesity; only a small proportion of patients had morbid obesity. We suppose that if the proportion of morbidly obese patients in our study was higher, the frequency and severity of postoperative complications would have been higher.

The present study was aimed at investigating the impact of BMI on early postoperative outcomes of patients undergoing lung lobectomy. The main limitation of the study is the retrospective and non-randomized design. Nevertheless, the study was adequately powered, took place in a single institution, involved consistent treatment management and surgical techniques were used in all patients.

## Conclusions

Obesity does not increase the incidence and severity of intraoperative and postoperative complications after lung lobectomy. Our study outcomes suggest that obesity paradox might be a reality in patients undergoing lung resection. However, further studies are required to offer reliable data regarding short-term outcomes of thoracic surgery in obese patients.

## Abbreviations

ASA: American society of anesthesiologists classification
BMI: Body mass index
FRC: Functional residual capacity
SSI: Surgical site infections
WHO: World health organisation
